# Research trends in biomimetic medical materials for tissue engineering: commentary

**DOI:** 10.1186/s40824-016-0053-7

**Published:** 2016-03-29

**Authors:** Ki Dong Park, Xiumei Wang, Jae Young Lee, Kyung Min Park, ShengMin Zhang, Insup Noh

**Affiliations:** Department of Molecular Science and Technology, Ajou University, 206 Worldcup-ro, Yeongtong-gu, Suwon, 16499 Korea; School of Materials Science and Engineering, Tsinghua University, Beijing, 100084 China; School of Materials Science and Engineering, Gwangju Institute of Science and Technology (GIST), Gwangju, 500-715 Korea; College of Life Science and Bioengineering, Incheon National University, Incheon, 22012 Korea; Advanced Biomaterials and Tissue Engineering Center, Huazhong University of Science and Technology, Wuhan, P. R. China; Convergence Institute of Biomedical Engineering and Biomaterials, Seoul National University of Science and Technology, Seoul, 01811 Korea; Department of Chemical and Biomolecular Engineering, Seoul National University of Science and Technology, 232 Gongneung-ro, Nowon-gu, Seoul, 01811 Korea

## Abstract

We introduce our active experts’ communications and reviews (Part II) of 2015 Korea-China Joint Symposium on Biomimetic Medical Materials in Republic of Korea, which reflect their perspectives on current research trends of biomimetic medical materials for tissue regeneration in both Korea and China. The communications covered three topics of biomimetics, i.e., 1) hydrogel for therapeutics and extracellular matrix environments, 2) design of electrical polymers for communications between electrical sources and biological systems and 3) design of biomaterials for nerve tissue engineering. The reviews in the Part II will cover biomimetics of 3D bioprinting materials, surface modifications, nano/micro-technology as well as clinical aspects of biomaterials for cartilage.

## Introduction

An invitation-based, bilateral symposium on biomimetic medical materials was held from October 22 to 26 in Seoul, Korea, towards building strong relationships among established leaders and emerging young scientists in Korea and China. Numerous breakthrough achievements in biomedical materials and nano-biotechnology research on regenerative medicine have been made in the two countries throughout successful series of the previous Korea-China Joint Symposium on Biomaterials and Nano-biotechnology pioneered by professors Inseop Lee in Yonsei University of Korea and Fu-Zhai Cui in Tsinghua University of China over more than 10 years.

Biomaterials science and technology have recently evolved into a new stage of tissue engineering research through the modeling of characteristics of the extracellular matrix (ECM) of defect tissues as well as the advancement of new fabrication methods of 3D bio-printings of functional biomaterials. The symposium focused on recent scientific progress on biomimetics and regenerative medical materials for tissue engineering, particularly bone, cartilage and nerve, and provided a platform for the academic cooperation between Korea and China.

During the discussion session about two countries’ scientific collaboration led by key scientists, diverse issues were mainly discussed on how to upgrade our scientific relationship of this new research area. Other topics also raised how to contribute to improve the relationships among the National Research Foundation (NRF) of Korea and the National Natural Science Foundation of China (NSFC), Korean Society for Biomaterials (KSBM) and Chinese Society for Biomaterials (CSBM) and how to improve partnerships in research and industrial aspects.

The key members of both Korean and Chinese Societies for Biomaterials agreed on holding a biannual exchange forum of young scientists during each society’s annual meeting. The first forum was held on November 20, 2015 in Hainan, China, and the second one is scheduled to be held in Korea in 2016.

Herein, we introduce our active experts’ communications and reviews, which reflect their perspectives on current research trends of biomimetic medical materials for tissue regeneration. The communications covered three topics of biomimetics, i.e., 1) hydrogel for therapeutics and extracellular matrix environments, 2) design of electrical polymers for communications between electrical sources and biological systems and 3) design of biomaterials for nerve tissue engineering. The reviews will cover biomimetics of 3D bioprinting materials, surface modifications, nano/micro-technology as well as clinical aspects of biomaterials for cartilage.

## Biomimetic medical materials trends: polymeric hydrogels

Polymeric hydrogels have received substantial attentions in various biomedical applications due to their structural similarities to the native extracellular matrix (ECM) as well as multi-tunable properties. Up to date, various kinds of polymeric hydrogels have been developed through physical- or chemical-cross linking mechanisms, which can be applied as either therapeutic implants or therapeutic vehicles for controlled drug delivery and tissue regeneration [1]. Specifically, the engineered polymeric hydrogels have been widely used either as therapeutic delivery carriers that facilitate tissue regeneration and repair or as an artificial extracellular microenvironment that support 3D cells or organs growth [2, 3]. Here, we briefly discuss the emerging trends in polymeric hydrogel materials for biomedical research fields.

### Therapeutic vehicles

In the past decade, polymeric hydrogels have been used as a carrier to deliver therapeutic agents (e.g., growth factors, cells or other bioactive molecules) for tissue regeneration [4]. Recently, researchers have focused on developing advanced hydrogel materials that allow much finer control over the spatial and temporal delivery of the therapeutic agents to improve the therapeutic efficacy. These engineered hydrogel materials can play a role not only as delivery vesicles for the therapeutic agents, but also to direct stimulate tissue regeneration and repair through physicochemical interactions between the materials and the host tissues. Park and his colleagues have developed a micro/nanogel composed of in situ crosslinkable gelatin-based microgel and self-assembled heparin-based nanogels, which can serve as an injectable growth factor delivery carrier for the urethral muscle regeneration [5]. Interestingly, they noticed that incorporating a growth factor -encapsulated heparin-nanogels into a gelatin matrix allowed the hydrogel composites to release the therapeutic agent continuously up to 4 weeks, resulting in enhanced urethral muscle regeneration and recovery of their biological function. More recently, Park and Gerecht have developed hypoxia-inducible hydrogels that can provide artificial hypoxic microenvironment when injected into the body, showing facilitating vascular tissue regeneration and tissue augmentation [6]. These innovative approaches have considerable values, and therefore hold great potential for tissue regenerative medicine.

### Artificial extracellular microenvironments

Recently, many researchers are interested in utilizing the artificial extracellular microenvironment using bio-mimetic hydrogel materials to support cell growth. These engineered cellular microenvironments are created by tailoring the polymeric backbone with cellular response molecules (e.g., cell adhesion peptides or proteolytic-cleavable peptides), which are critical for supporting 3D cell growth [7]. In addition, it is well known that the native cellular microenvironment contains spatial-gradients in various physicochemical properties, including matrix proteins, oxygen gradients, mechanical strength, and microstructure properties [8, 9]. Combining these parameters, the synthetic microenvironments have been utilized either to create three dimensional tissue constructs for tissue regeneration or to generate engineered disease models (e.g., engineered tumor, vascular, skin, and liver models) for a better understanding of basic cellular/molecular biology and clinical outcomes. More recently, the engineered tissue constructs have been designed by a combination of polymeric hydrogels with micro-/nano-fabrication techniques (e.g., microfluidics and 3D printing) to more accurately recapitulate the complexity of the native cellular environments [10–12].

## Biomimetic medical materials trends: design of electrical polymers for biomimetics

Tissues and cells in our body are strongly affected by electrical signals, such as electrical field and current, and at the same time utilize electrical signals as important factors that control physiological conditions [13]. For example, embryonic body development and tissue regeneration are known to involve electrical signals [14]. The simple examples include electrically excitable cells, such as neurons, cardiomyocytes, myoblasts, utilize electrical signals for the inherent functions [15]. Surprisingly, recent studies have revealed that other types of cells, such as stem cells, also respond to electrical stimulation and exhibit various cell behaviors [16]. Accordingly, there have been growing attentions to an effective way to deliver or record electrical signals to or from biological systems. To effectively mediate electrical signals between electrical sources (e.g., electrodes) and biological systems, materials that possess good electrical conductivity are required. Various conductive materials, including metals, metal oxides, carbon nanomaterials, and conductive polymers, have been extensively utilized to construct conductive interfaces. Among them, electrically conductive materials, polypyrrole (PPy), polythiophene (PT), and PEDOT, are conductive organic materials [17]. Compared to other conductive materials, these conductive polymers offer some important advantages, such as biocompatibility, ease in modification, lower rigidity, and redox activity. Consequently, these conductive polymers have been widely used as a whole materials or components in tissue scaffolds, sensors, biolectrodes, and so forth.

Native tissue and its environments are highly organized and their properties are harmonized to permit cues modulating biological activities. Depending on the physiological and pathological states, native environments actively respond and dynamically change their composition, structures, and bioactivities [18]. Therefore, profound effects have been made to incorporate the properties of the native environments such as ECM into biomaterials to create ‘artificial’ environments, of which strategy is known as ‘biomimetics’. Likewise, electrically conductive polymers can be tailored to delivery additional cues that the native tissues present. This approach will benefit the production of effective materials for a wide range of applications, such as regenerative medicine and long-term biocompatibility of the implants. In addition to electrical activity, important factors to be considered in fabricating biomimetic conductive biomaterials include mechanical rigidity, structures, and bioactivities. Note that biomimetic conductive materials should be designed and fabricated to achieve the best responses depending on the target biological system.

First, in comparison with the rigidity of conductive polymers (> MPa), the most tissues are softness with a few Pa (brain tissues) to kPa except bone [19]. This mechanical mismatch not only causes poor direction of cellular responses but also inflammatory tissue reactions [20]. For example, cells and tissues, especially stem cells, generally exhibit the fast and specified growth and differentiation when cultured with the materials present similar mechanical properties to the native tissues [21]. Hence, this indicates the needs of developing softer and flexible conductive materials. To this end, composite hybrid materials were fabricated by mixing or growing conductive polymers with elastic materials or hydrogels. Interestingly, conductive hydrogels can mimic the mechanical softness of the native soft tissues by presenting tens of kPa of Young’s modulus [22]. Yet, lowering the rigidity accompanies the impairment of electrical conductivities, which has to be overcome in the future studies. Still, since some biomedical applications need small currents, the conductive hydrogels will be useful.

Another important property is a structure, which can serve as biomimetic features of extracellular matrices including porosity, nano-/micro-structures, and orientation [23]. Advances in technologies enable the fabrication of such structurally effective conductive materials, which allow for the production of biologically important subcellular scaled features. For example, electron beam lithographic patterns of PPy could facilitate the polarization of embryonic hippocampal neurons [24]. Also, various fibrous structures of conductive polymers could be obtained by direct electrospinning, phase separation, and nano-coating of nanofibrous mats [25]. Interestingly, recent studies done by Hardy et al. demonstrated the enhanced osteogenesis of human mesenchymal stem cells cultured on PPy-silk nanofibers by electrical stimulation [26], suggesting the cooperative or additive roles of topographical cues and electrical cues as biomimetic functional materials. Likewise, a variety of conductive nanofibers have been produced for different types of cells.

Lastly, various biological active molecules are to be immobilized in/or conductive polymers by physical or chemical fashions. Since the effect interactions are often mediated by receptor-ligand binding, it will be critical to fabricate biologically active conductive materials by immobilizing ECM proteins, polysaccharides, and growth factors. In particular, anioinic proteins and mucopolysaccharides can be doped into oxidized conductive polymers, which can be easily produced during polymerization processes of conductive polymers [27]. Covalent immobilization of ECM proteins and growth factors enables prolonged interactions with cells without substantial consumption and signal regulation via binding with integrins and tyrosine kinase receptors, respectively. These signaling pathways finally affect gene expression levels of various genes related with proliferation, survival, and differentiation. For example, nerve growth factor immobilization onto conductive polymers have been attempted to enhance neural cell differentiation [28]. Interestingly, growth factors and electrical stimulation through conductive scaffolds could act together to induce the neurite formation and elongation. In addition, various ECM-derived peptides (e.g., Arg-Gly-Asp) were also incorporated into conductive polymer scaffolds to better mimic the native ECM, which could support attachment, growth and differentiation of various cells, such as neural cells [29], endothelial cells [30], cardiac cells [31], and fibroblasts [32]. For example, cell membrane mimicking conductive polymer having IKVAV (Ile-Lys-Val-Ala-Val) peptide and phosphorylcholine promoted neurite outgrowth and protein secretion on neural cells [33].

In summary, biomimetic conductive polymers can offer great promise to actively modulate biological systems. Still, better mimicry of characteristics of the target tissues and their demonstration will be desirable. High performance conductive polymer-based biomaterials will be greatly beneficial for applications of tissue engineering, drug delivery, and biocompatible bioelectrodes.

## Biomimetic medical materials trends: design of biomaterials for nerve tissue engineering

The design of optimal biomaterials for regenerating injured human tissues and organs plays a crucial role in tissue engineering and regenerative medicine, especially for highly complex nerve system [33, 34]. Biomimetics, a science of mimicking natural phenomena of a biological system in terms of its composition, structure and function as a model for engineering new materials and systems, has been a promising strategy for inspiring the design and fabrication of novel biomaterials for tissue engineering and regenerative medicine. From the viewpoint of biomimetics, new generation of biomaterials should serve as not only structural support, but also artificial microenvironments that mimic the natural stem cell niches to deliver stem-cell-regulatory signals to purposefully stimulate specific cellular responses at the molecular level for mediating the regeneration of living tissues with full restoration of normal structure and function [33–35]. Biomaterial properties, such as the material architecture, mechanical properties, surface topographies, chemical properties, and biological ligands have been widely proven to exert influences as regulatory cues on stem-cell fate both in vivo and in vitro.

Previous studies indicate that biophysical properties of biomaterials are very important design parameters in preparing artificial regenerative niche for nerve regeneration. For example, the stiffness/elasticity of the underlying substrate could direct stem cell fate and even regulate their lineage differentiation [36, 37]. Adult neural stem cells exhibited promoted neuronal differentiation on soft substrates (100–500 Pa), while stiffer substrates (1,000–10,000 Pa) led to glial differentiation. Besides, it has been suggested that mesenchymal stem cells (MSCs) fate can also be directed by matrix elasticity of 0.1 ~ 1 kPa toward neural differentiation. Therefore, the biomaterials designed for neural tissue regeneration should have lower stiffness to mimic the soft ECM of the neural tissue, which has positive effects on spontaneous neural differentiationof endogenous and/or exogenous stem cells. Furthermore, it has been widely accepted that aligned microstructures of biomaterials are of vital importance in nerve tissue engineering based on biomimetic ideas. Natural nerve tissues, not like most of other living tissues, have hierarchically oriented structures from a single neural axon to nerve fibers that are closely related to the directional transmission of nerve impulses. Previous studies have shown that the aligned structure was extensively identified in designing nerve conduits due to their ability of promoting alignment and elongation in regenerating axons, and regulating preferential neural differentiation of stem cells [38–40]. Beyond that, aligned fibrous structures provided a topographical cue to stimulate the elongation and enhanced neuronal differentiation of both adult neural stem cells [41] and MSCs [42] compared to random fibers. However, most of the previous studies applied 2-dimensional (2D) aligned fibrous membranes or meshes in cell culture that actually have a prominent difference on the stiffness of nerve tissues. Therefore, a suitable biomaterial for nerve regeneration should be soft with lower elasticity resembled natural neural matrix, and at the same time has 3-dimensional (3D) aligned structures.

In our study, a hierarchically aligned fibrillar fibrin hydrogel that was fabricated through electrospinning and the concurrent molecule self-assembly process mimics the soft and oriented features of nerve tissue simultaneously, thus providing hybrid biophysical cues to instruct cell behavior in vitro and in vivo. We found that the low elasticity and aligned topography of AFG had co-effects on promoting the neurogenic differentiation of human umbilical cord mesenchymal stem cells (hUMSCs), and also inducing dorsal root ganglions neurons to project numerous long neurite outgrowths longitudinally along the fibers rapidly for total migration distance of 1.96 mm in three days without the supplement of neurotrophic factors. Moreover, the aligned fibrin hydrogel implanted in a rat T9 dorsal hemisection spinal cord injury model was found to promote endogenous neural cells fast migration and axonal invasion along the fibers constructing aligned tissue cable in vivo. Our results suggest that matrix stiffness and aligned topography could synergistically instruct stem cell neurogenic differentiation and rapid neurite outgrowth, providing great promising in biomaterials design for applications in nerve regeneration.
